# Establishment of a Low-Level Laser Irradiation Protocol for *In Vitro* Development of Bovine Embryos

**DOI:** 10.3390/vetsci13090913

**Published:** 2026-09-05

**Authors:** Aleksandar Plavšić, Minja Zorc, Peter Dovč

**Affiliations:** 1 Biotechnical Faculty, University of Ljubljana, Jamnikarjeva 101, 1000 Ljubljana, Slovenia; ap2102@student.uni-lj.si (A.P.); minja.zorc@bf.uni-lj.si (M.Z.); 2 Vets4science d.o.o., Kukovčeva Ulica 2, 3000 Celje, Slovenia

**Keywords:** cattle, in vitro embryo production, photobiomodulation, low-level laser, blastocyst rate, embryo transfer

## Abstract

Photobiomodulation using low-level laser irradiation has been proposed as a non-thermal approach for stimulating cellular metabolism, but practical protocols for its application to bovine embryos after fertilisation remain insufficiently described. This study aimed to establish a practical low-level laser irradiation procedure at different time points during the in vitro development of bovine embryos. Abattoir-derived oocytes were matured, fertilised with semen from a single bull, and cultured under standard conditions. Entire culture dishes were irradiated using a K-Laser Cube at 800 nm and 0.10 W for 200 s, delivering 20 J, on day one, three or five after fertilisation; an untreated run served as a reference. Embryos were evaluated on day seven according to International Embryo Transfer Society standards. Across four separate production runs, 1345 inseminated oocytes yielded 348 blastocysts meeting IETS stage 6, quality grade 1 criteria (26% overall); blastocyst rates were 27.8%, 22.0%, 25.9% and 28.5% for the reference, day one, day three and day five runs. Because each treatment was tested in a separate run, these values are descriptive and do not permit assessment of treatment efficacy. The procedure was incorporated into routine bovine in vitro embryo production and provides a basis for future validation studies.

## Simple Summary

Low-level laser irradiation has been proposed as a non-thermal method that may influence cell activity during embryo development. In this study, we established a protocol for applying low-level laser irradiation to bovine embryos at different time points after fertilisation during routine laboratory production. Across four separate production runs, 1345 inseminated oocytes yielded 348 blastocysts meeting IETS stage 6 quality grade 1 criteria. Because each irradiation condition was tested in a different production run, the study design did not allow a conclusion whether irradiation improved embryo development. The protocol provides a practical basis for future studies of treatment efficiency.

## 1. Introduction

In vitro embryo production (IVP) and embryo transfer (ET) are established reproductive biotechnologies in cattle breeding, allowing more efficient use of the genetic potential of valuable female animals [1,2]. Despite substantial improvements in laboratory procedures, IVP efficiency remains inherently variable: typically, only 30–40% of fertilised oocytes reach the blastocyst stage, and in vitro-produced embryos differ from their in vivo counterparts in quality, cryotolerance and pregnancy rate after transfer, thereby maintaining interest in non-invasive interventions that can be incorporated into routine embryo culture without disrupting the established laboratory workflow [3,4,5].

Low-level laser irradiation (LLLI), also described in the literature as photobiomodulation (PBM), following an international nomenclature consensus that recommended this broader, mechanism-based term [6], uses low-power red or near-infrared light to elicit biological responses without inducing a significant thermal effect. Its activity is attributed primarily to absorption by mitochondrial chromophores, in particular cytochrome c oxidase, which can transiently increase electron transport, ATP synthesis and redox signalling, thereby modulating cell proliferation and survival. Importantly, the biological response to LLLI is not linear: PBM effects typically follow a biphasic, dose-dependent pattern in which sub-threshold doses have no measurable effect, an intermediate range is stimulatory, and excessive doses become inhibitory [7,8]. The dependence of PBM on wavelength, power, energy density, exposure time and target tissue means that outcomes cannot be generalised across studies unless irradiation parameters are reported in full and can be reproduced [9].

Evidence for LLLI/PBM effects on reproductive cells and early embryos is accumulating but remains heterogeneous. In cattle, irradiation applied during the oocyte maturation altered the metabolic profile of oocytes and embryos without increasing the blastocyst yield [10], whereas irradiation of superovulated donor cows was associated with a higher number of recovered embryos—a result of an in vivo intervention that differs substantially from the direct irradiation of embryos in culture [11]. More recently, PBM applied during the in vitro maturation of bovine oocytes was reported to increase ATP content and support subsequent embryonic development [12]. Positive effects on preimplantation embryo development have also been described in mice [13].

Differences between species, developmental stage, irradiation parameters and treatment timing, however, limit direct comparison among studies and hamper its translation into a standard protocol. Detailed practical protocols for applying low-level laser irradiation directly to bovine embryos at defined post-fertilisation time points with integration into routine in vitro embryo production workflows are scarce [9]. Therefore, this study aimed to establish a reproducible procedure for low-level laser irradiation of entire culture dishes on days 1, 3 or 5 after fertilisation during routine bovine in vitro embryo production, with day-7 blastocyst development recorded as a descriptive indicator of feasibility of the procedure.

## 2. Materials and Methods

Embryos were produced in routine weekly in vitro production (IVP) runs following the manufacturer’s bovine IVF commercial protocol (IVF Bioscience, Falmouth, UK) [14], with the modification that 200 µL medium drops were used throughout. In separate production runs, embryos were either not irradiated (reference run) or irradiated once on day 1 (L1), day 3 (L2) or day 5 (L3) after fertilisation. Each condition was represented by one independent IVP run. The preparation of media, dishes and handling were performed in a sterile laminar-flow hood.

**Figure 1:**
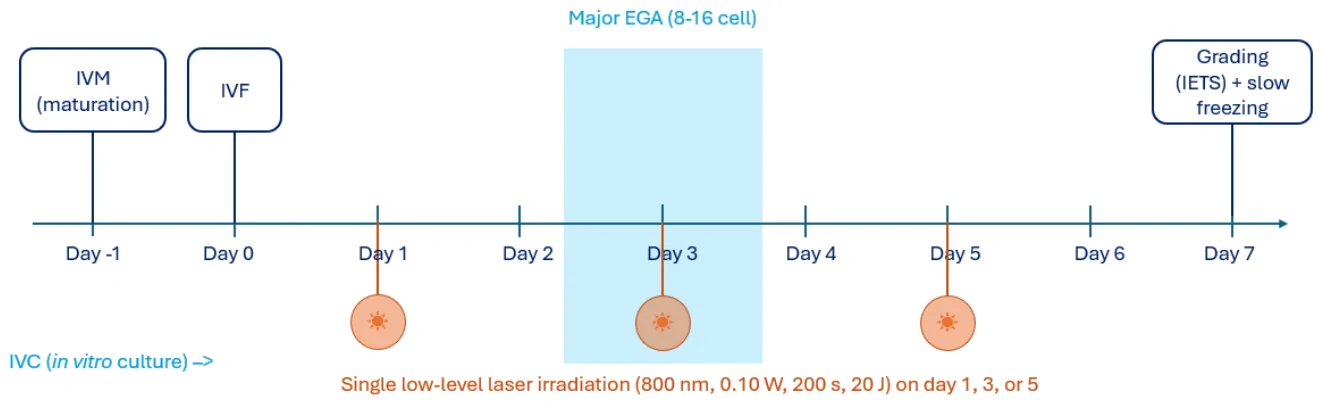
Timeline of in vitro embryo production and the three low–level laser irradiation time points.

The overall workflow and the three irradiation time points are shown in Figure 1.

Bovine ovaries were obtained from a commercial abattoir and transported to the laboratory at 20–25 °C within approximately 10–15 min. Cumulus–oocyte complexes (COCs) were aspirated from follicles with a diameter of 2–6 mM using an 18 G needle and a 12 mL rubber-free syringe. All culture and handling media were from the BO-range (IVF Bioscience, Falmouth, UK) and were used according to the manufacturer’s protocol; media were prepared at least 2 h in advance and equilibrated in the incubator. COCs were matured in BO-IVM medium in groups of ≤20 per 200 µL drop (four drops per dish) at 38.8 °C in humidified air containing 5.5% CO_2_ for 21–24 h, in an Esco Celculture CO_2_ incubator (model CCL–050B–8–IVF; Esco Micro, Singapore).

Frozen–thawed semen from the same batch of a single bull was used for all runs. Semen was thawed and prepared in BO-SemenPrep; sperm motility and concentration were determined, and a motility-compensated volume of sperm suspension was added to each 200 µL fertilisation drop to reach a final concentration of approximately 2.0 × 10^6^ motile spermatozoa/mL. The per-run motility, sperm concentration, and insemination doses are presented in Table 1. Fertilisation was performed in 200 µL BO-IVF drops with ≤20 oocytes per drop and incubated for approximately 16–20 h at 38.8 °C in humidified air, containing 5.5% CO_2_ (Esco Celculture CCL-050B-8-IVF).

On day 1 after fertilisation, cumulus cells were removed by vortexing at 2600 rpm for approximately 2 min 30 s to 2 min 45 s (MX-S vortex mixer; DLAB Scientific, Beijing, China), followed by washing in BO-Wash medium (IVF Bioscience, Falmouth, UK) with concurrent denudation using a 135 µm denudation pipette (Pipet-ID; Gynetics Medical Products, Lommel, Belgium) mounted on a micropipettor. Presumptive zygotes were cultured in BO-IVC medium in groups of ≤ 20 per 200 µL drop (four drops per dish) under mineral overlay oil (IVF Bioscience, Falmouth, UK) for 7–9 days in a humidified tri-gas atmosphere of 5.5–6.5% CO_2_, 6% O_2_ and 87.5–88.5% N_2_, in an Esco Celculture CO_2_/O_2_ incubator (model CCL-050T-8-IVF; Esco Micro, Singapore).

**Figure 2:**
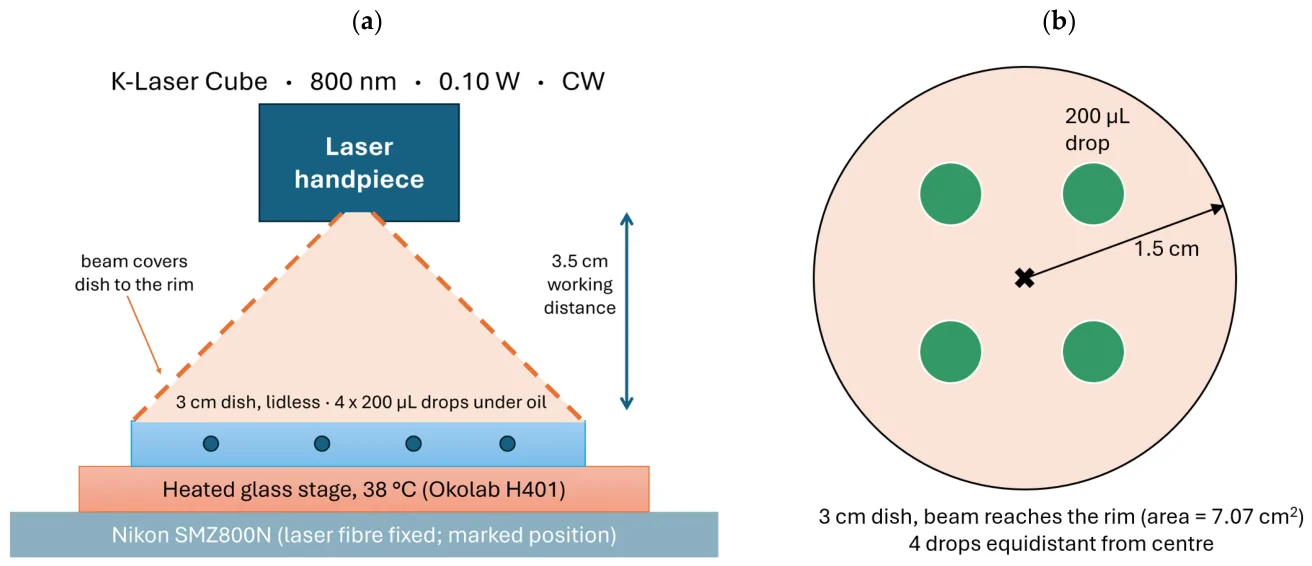
Low-level laser irradiation setup: (**a**) side view and (**b**) top view of the culture dish during irradiation.

Irradiation was performed using a K-Laser Cube device (Eltech K-Laser S.r.l., Treviso, Italy) operating at a wavelength of 800 nm in continuous-wave mode, at a power output of 0.10 W for 200 s, delivering a total energy of 20 J per exposure. The irradiation setup is shown in Figure 2.

The laser fibre-optic cable was fixed to a Nikon SMZ800N stereomicroscope (Nikon, Tokyo, Japan) so that the handpiece was suspended vertically above the dish; the attachment points on both the fibre and the microscope were permanently marked to reproduce the same geometry at every session. The handpiece aperture was positioned 3.5 cm above an uncovered (lidless) 3 cm diameter plastic Petri dish; at this distance the beam covered the dish exactly to its rim, corresponding to an irradiated area of ≈7.07 cm^2^. The delivered dose therefore corresponded to an irradiance of ≈14 mW/cm^2^ and a fluence of ≈2.8 J/cm^2^ across the dish.

Each dish contained four 200 µL medium drops (holding oocytes for maturation, or fertilised oocytes and developing embryos), arranged equidistantly around the centre of the dish so that all four drops were at an equal radial distance from the beam axis and received equivalent light scattering. Drops were overlaid with 3.5–4 mL of mineral overlay oil (IVF Bioscience, Falmouth, UK) to ensure complete coverage. During irradiation, dishes rested on a heated glass stage set calibrated to 38 °C maintained by an H401-T-Controller (H401 series; Okolab, Pozzuoli, Italy) fitted to the same Nikon SMZ800N stereomicroscope. Each embryo group received a single irradiation on its assigned time point. The non-irradiated reference run was handled identically—placed on the heated stage with the lid open for 200 s—but was not exposed to the laser. The same microscope was used for cumulus removal, embryo manipulation and morphological evaluation.

The irradiation parameters were selected on the basis of previously described photobiomodulation parameters. The three time points were selected to set irradiation at distinct developmental windows relative to embryonic genome activation (EGA): day 1 (pronuclear/early cleavage, before major EGA), day 3 (around and after the major bovine EGA at the 8–16-cell stage [15]) and day 5 (morula/early blastocyst stage).

The embryos were grown to day 7 and morphologically graded according to IETS criteria [16]. Only embryos classified as developmental stage 6 and quality grade 1 were included in the blastocyst yield reported in this study.

All laboratory procedures were recorded on the bovine IVP mate sheet during embryo production and handling.

## 3. Results

**Figure 3:**
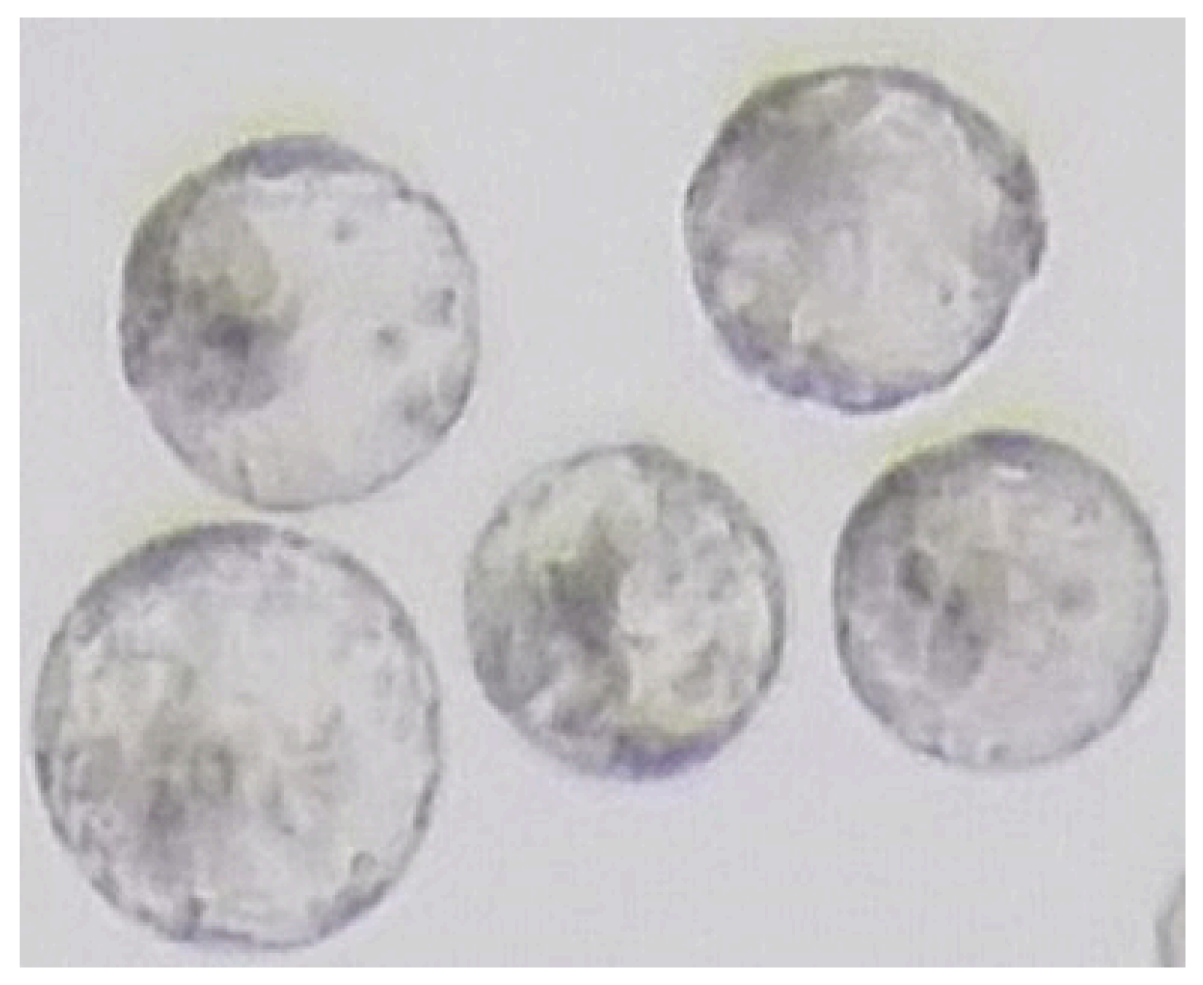
Embryos on day 7 which were classified as developmental stage 6 and quality grade 1, according to IETS criteria.

Four routine IVP runs were performed, one for each condition (non-irradiated reference and irradiation on day 1, day 3, or day 5 after fertilisation). Across all runs, 1345 inseminated oocytes yielded 348 IETS stage 6, quality grade 1 blastocysts (Figure 3), corresponding to an overall blastocyst rate of 25.9%. Successful development of blastocysts was obtained in every run (Table 2).

The non-irradiated reference run yielded 65 IETS stage 6, quality grade 1 blastocysts from 234 inseminated oocytes (27.8%). The day 1, day 3 and day 5 runs yielded 79 of 359 (22.0%), 101 of 390 (25.9%) and 103 of 362 (28.5%) IETS stage 6, quality grade 1 blastocysts, respectively).

Since each irradiation treatment was implemented in a separate production run, irradiation timing and production batch were confounded; the blastocyst rates are therefore reported as descriptive outcomes and were not subjected to inferential statistical comparison.

## 4. Discussion

Low-level laser irradiation was successfully integrated into the routine in vitro embryo production workflow, with blastocysts obtained in all four production runs and an overall blastocyst rate of 25.9%. In the present study, ovaries were recovered from a different, genetically and physiologically heterogeneous group of abattoir-slaughtered donors for each production run. Donor background has been shown to influence the in vitro development of abattoir-derived bovine oocytes [17]. Repeated ovum pick-up studies have demonstrated a persistent oocyte donor effect in which the best and the worst blastocyst producers remained the same animals, irrespective of the semen used [18]. Laboratory setup and handling can have an effect as well: site-dependent differences in blastocyst yield have been reported even when semen and biological extracts were shared between production sites [19]. Differences in blastocyst rate between production runs are therefore expected in bovine embryo production and do not by themselves indicate a treatment-specific effect of irradiation in our experiment.

Blastocysts developed in all irradiated production runs; however, because treatment conditions were confounded with production batch, the present study cannot determine whether irradiation had an effect on embryo developmental competence. However, compatibility should not be equated with biological benefit. The current design cannot distinguish the effect of irradiation from the effect of the production batch because embryos from the same batch were not randomised between irradiated and non-irradiated conditions. Accordingly, the present data do not demonstrate an increase in the yield of IETS stage 6, quality grade 1 blastocysts attributable to LLLI. Future validation experiments should therefore include an untreated or sham-treated control within every production run, with oocytes or presumptive zygotes from the same batch randomly allocated among treatment conditions.

The irradiation setup is reported in detail to facilitate replication and minimise variation in positioning, exposure time and temperature. Nevertheless, the reported irradiance and fluence represent calculated nominal values based on the output power and the illuminated area. Unless optical power was measured directly at the level of the culture dish, the actual energy reaching the embryos may have differed because of beam non-uniformity and variation in culture medium and mineral oil volume. Direct measurement of power at the sample plane, together with mapping of beam uniformity across the dish, would strengthen future validation of the procedure. The temperature should also be monitored during irradiation to confirm that the observed effects remain photobiomodulatory rather than thermal.

The three irradiation time points were selected to represent biologically distinct stages of preimplantation development. Day 1 represents the pronuclear or early cleavage period, day 3 approximately corresponds to major bovine embryonic genome activation [15], and day 5 represents the morula or early blastocyst stage. It is biologically plausible that embryos could respond differently to photobiomodulation at these stages because mitochondrial activity, cell number, and transcriptional control change substantially during early development. The present descriptive data do not indicate a clear optimal time point because the variation between production runs was considerable and the number of independent runs per condition was limited.

Future studies should use a randomised, within-batch design in which oocytes or embryos from each production run are divided among a sham-treated control and the different irradiation schedules. Multiple independent runs should be included for every condition, and the operator should ideally be blinded during embryo grading. In addition to blastocyst rate, evaluation should include cleavage rate, developmental kinetics, blastocyst quality, cell number, mitochondrial activity and post-cryopreservation survival. Given the biphasic dose–response characteristic of photobiomodulation, in which insufficient doses may have no effect and excessive doses may become inhibitory [7,8], one possible explanation for the similar blastocyst yields is that the selected irradiation dose was not within an optimal stimulatory range. Future dose–response studies comparing several fluence levels would therefore be valuable.

In summary, the present work provides a practical protocol for applying LLLI at different stages of embryo culture. Blastocysts developed following irradiation at all evaluated time points.

## 5. Conclusions

A practical low-level laser irradiation procedure was successfully incorporated into routine bovine in vitro embryo production and applied on day 1, day 3 or day 5 after fertilisation. Embryos developed to the blastocyst stage following irradiation at all evaluated time points. Because irradiation conditions were tested in separate production batches, the observed blastocyst rates cannot be attributed specifically to the laser treatment. The protocol provides a reproducible treatment as a basis for future randomised, within-batch studies designed to evaluate biological efficacy, optimal treatment timing and dose–response relationships.

## Figures and Tables

**Table 1:** Semen motility, concentration, and insemination doses per production run. All runs used frozen–thawed semen from the same batch of a single bull.

Condition	Motility (%)	Concentration (×10^6^/mL)	Standard Dose (µL)	Motility-Compensated Dose (µL)
No irradiation	45	100.0	4.0	8.9
Day 1	40	120.0	3.3	8.3
Day 3	55	105.0	3.9	8.6
Day 5	45	111.7	3.6	8.7

**Table 2:** Day 7 yield of IETS stage 6, quality grade 1 blastocysts by irradiation treatment.

Condition	Inseminated Oocytes	Blastocysts (IETS Stage 6, Quality Grade 1)	Blastocyst Rate (%)
Non-irradiated (reference)	234	65	27.8
Day 1	359	79	22.0
Day 3	390	101	25.9
Day 5	362	103	28.5

## Data Availability

The original contributions presented in this study are included in the article. Further inquiries can be directed to the corresponding author.

## Abbreviations

The following abbreviations are used in this manuscript:

ATP	adenosine triphosphate
COC	cumulus–oocyte complex
EGA	embryonic genome activation
ET	embryo transfer
IETS	International Embryo Transfer Society
IVF	*in vitro* fertilisation
IVM	*in vitro* maturation
IVP	*in vitro* embryo production
LLLI	low-level laser irradiation
PBM	photobiomodulation
